# Neuralgia

**Published:** 1875-07

**Authors:** R. L. Cochran

**Affiliations:** Burlington


### NEURALGIA.

BY R. L. COCHRAN, BURLINGTON.

*Read before the Iowa State Dental Association.*

*Mr. President and Gentlemen:*—I wish to occupy a short time upon a subject with which I claim every dentist should be familiar; for how often are we called upon to relieve pain with which the teeth have no connection, and though there are many diseases of the mouth and head which should come under the treatment of the dental physician, yet I shall confine myself for the present to neuralgia, a disease of the nervous system manifesting itself by pains nearly always unilateral which appear to follow the course of particular sensory nerves; the pains are usually sudden, of a darting, boring or burning character; they are at first unattended with any local change which can be recognized; it is universally the case that the existing condition of the patient at the time of the first onset of the disease is one of debility, either special or general, so that the attention of the physician must not be directed only to controling the pain but also to remove the cause, whatever that may be. Usually the dentist is not sonsidered as having the authority to treat his patient beyond the teeth, and I am sorry there are some dentists who so regard the matter themselves, and yet consider they are in the way of progress; but, friends, believe me, the time is not for distant when the intelligent and educated dentist will be required to treat his patient, no matter whether the cause be in the head or stomach, and I say further, the dentist who is unable to write an intelligent prescription, not alone for neuralgia, but the many other diseases which are constantly presented, is far behind the standard of a first class dentist. I ask, why send your patient to an M. D. when it requires something more than filling or extracting a tooth? Are they of a different creation? No, gentlemen, they are not; then let us prepare ourselves to treat our patients who place themselves under our care, without asking aid of those whe become acquainted with the disease only as we can, for I repeat it, we can not be successful dentists and rise to the standard of our blessed hope, and yet confine ourselves to putting carbolic acid in a decayed tooth. With this feeling I wish to offer a few suggestions relative to neuralgia, knowing it is too complicated a disease to do more at this time than simply touch a few points.

A great characteristic of neuralgic affections is that the pain is intermittent (or, at least, remittent) in every stage of the disease. One of the most common varieties of neuralgia, is sick headache; the affection is entirely independent of digestive disturbances, though it may be aggravated by their occurence; it usually attacks persons during their bodily development, induced my precocious straining of the mental powers, suffering most after fatigue or excitement, the patient generally finding relief in nausea and vomiting.

Neuralgia may be be ushered in by a remarkable anæthestic condition of the parts, afterwards to become very painful. Severe attacks of neuralgia are usually complicated with secondary affections or other nerves, and in this way congested blood vessels, arrested secretions from glands, ulceration of tissue, etc. are sometimes brought about. I knew of a case, where a piece of iron being removed from the arm cured neuralgia in the head after almost every known remedy had been given without any good effect, save temporary relief from pain.

It is also noticeable that with a general declination of malarial fevers, consequent on improved drainage or cultivated lands that neuralgia becomes constantly more scarce. Neuralgia is oftener met with in persons between 25 and 45 years of age than younger or older, for it is in this time of life that individuals are subjected to the most serious pressure from external influences; the men if poor, are struggling for the maintenance of their families, or if rich and idle are immersed in dissipation, while the women are going through the exhausting process of child bearing, or are devoured with anxiety for a certain position in fashionable society, but the most serious time to be attacked with neuralgia is the period of declining life. It is at this time most severe and intractable, and but little encouragement can be given persons after they have entered upon, what may be called the degenerated period of existence.

Neuralgia is eminently hereditary. It prevails in particular families, breaking out in successive generations, and is claimed by some, that these neuralgic families are distinguished by a tendency to paralysis, epilepsy, hypochondriosis or alcoholic excess, and it is a fact that certain influences, especially that of excessive drinking, which tend to produce degeneration of the nervous centers, are powerful predisposers to neuralgia showing that the descendents of drunkards often suffer the pain of the father's sin.

Anæmia (or the diminution of red globules) by whatever cause induced, favors the occurence of neuralgla, for a very large proportion of those who suffer from neuralgia are anæmic, with regard to the dependence of neuralgia on anœmia, Romberg says, it seems as if pain were the prayer of the nerve for healthy blood. Under the head of neuralgia are braced a group of local affections characterized by pain occurring without inflammation or any appreciable change in the part affected. All that can be said of the pathological character of these affections, is that they consist in a perversion of sensibility, it may be abruptly developed, but, in the majority of cases, there are premonitions. I believe it is generally conceded to be sudden in one-seventh and gradual in six-sevenths of cases, the premonitions consist of a feeling of weight or dull pain or a sense of heat, or some uncomfortable sensation in the seat of the affection.

There are two functions always wrong; those of the nervous system on the one hand, and those of the digestive system on the other. You must seek to put the digestive organs right, or to soothe the nervous system, according as they may seem to be the principal. Neuralgia becomes less curable in each successive decade of life, more especially after the commencement of organic degeneration. The diagnosis of neuralgia is to be based on the character of the pain, its situation in the course of the nervous trunk or its branches, tenderness of the parts, shifting of pain from one nerve to another; thus the neuralgia may be transferred from a cranial to a spinal nerve.

On the whole, the diagnosis of neuralgic affections, from others which may involve pain, is not so difficult, if it is possible to get a full account of the patient's history. The points to be considered are whether the pain is unilateral, has the patient suffered from neuralgia before? and if not, has it prevailed in his family? Was it preceded by numbness, or nervous depression? whether the excitant appeared to be cold, or a nervous shock, or a direct physical injury? Has the pain continued any length of time? Whether it is not dependent on a a local, mechanical, leach as a tumor, etc., are there any diseased teeth? An affirmative answer to any two or three of the questions, will justify treatment for neuralgia.

The treatment of neuralgia depends upon the improvement of the general nutrition, that of the nervous system, proper condition of the blood, narcotic stimulants, and remedies which exert a direct influence upon the affected nerve, and may be classified under three heads. The first division includes all remedial measures which are intended to improve the nutrition, particularly that of the nervous system, or to remove any vicious condition of the blood which may impair nervous functions. The second division includes the narcotic stimulant remedies. The third division comprises all the remedies which are destined to exert a direct influence upon the affected nerve.

The constitutional treatment under the head of nutritive remedies deserves our first attention. By far the most important is the animal fats, for fat must undoubtedly be applied to the nutrition of the nervous system. Cod liver oil occupies the highest rank among fatty remedies, and should be given where it is agreeable to the stomach, in doses of one table spoonful, two or three times a day, alone or mixed with some mucilaginous liquid, and in connection with this for anæmia, would give prec. carb. of Iron in doses of 5 to 30 grains two or three times a day.

Where there is a rheumatic diathesis, iodide of potassium five to ten grains twice a day, acts very favorably, and as an anti-periodic, quinine in three to ten grs. doses as often as required. As a narcotic stimulant remedy there can be no doubt of the power of alcohol, it is as distinct as that of opium, but the dangers of prescribing it as a remedy are too evident, for often instead of employing it in the moderate stimulant doses, which really are of service, they drown their pain with a large narcotic dose, and thus contract a liking for the oblivion of drunkenness. I am nevertheless convinced from the opinions of such men as Radcliff and Begbie, that a fixed daily allowance of not more than one ounce is a decided help to the recovery from every form of neuralgia, there should also be administered hyoscyamus, coninm, aconite, morphia, etc., either by the use of the hypodermic syringe or stomachic doses. The advantage gained by the use of the hypodermic over the administration by the mouth, are the greater promptness with which relief is obtained, the small quantity of medicine required, and the less interference with the digestive functions, and in many cases the avoidance of unpleasant after effects of an opiate. I believe it is thought best to use morphia or chloroform with the hypodermic, and not to use more than one-eight of a grain of morphia for males and the one-sixteenth of a grain for females, and not more than three to 6 minims of chloroform allowing sufficient time between injections to judge of the narcotic effects.

As regards the remedies used in the third division. According to the conclusions of Dr. Valliex none compares with blistering in value; the application if made to the foci of pain excites a direct stimulant effect upon the painful nerve, solacing even when they do not cure, the actual cautery has been used as a counter irritant although dry cupping and sinapisms are more general. There are a number of so called specific remedies for neuralgia, but as it depends upon different causes in different persons, it is absurd to expect that any single drug or even any one plan of treatment will always remove it. We must investigate all the particulars of the case and prescribe accordingly, for there are many cases so obstinate that it would require months to remove, while others are so simple that a teaspoonful of carbonate of soda dissolved in a little water, taken into the stomach will afford immediate relief by causing carbonic acid to be created, other times a simple purgative will be all that is required. Persons predisposed to neuralgia should always avoid exposure to cold and damp air, without sufficient clothing, thick veils for the face and flannel under clothing are very important as direct remedies and as regards mental influences which unfortunately are often beyond our control, one can only say that the two extremes of a specially laborious and exciting life and an existence spent in the dreamy monotony of idleness are equally hurtful. I have not mentioned the neuralgia so often caused by dead and diseased teeth, exostosis, etc., because I am of the opinion, gentlemen, you are aware of these, and know the treatment.

